# Dual targeting improves capture of ultrasound microbubbles towards activated platelets but yields no additional benefit for imaging of arterial thrombosis

**DOI:** 10.1038/s41598-017-15080-7

**Published:** 2017-11-02

**Authors:** F. Günther, T. Heidt, M. Kramer, E. Khanicheh, A. L. Klibanov, A. Geibel-Zehender, E. A. Ferrante, I. Hilgendorf, D. Wolf, A. Zirlik, J. Reinöhl, C. Bode, K. Peter, B. A. Kaufmann, C. von zur Mühlen

**Affiliations:** 1 Heart Center Freiburg University and Faculty of Medicine, University of Freiburg, Freiburg, Germany; 2 University Hospital of Basel, Basel, Switzerland; 3 University of Virginia, Charlottesville, USA; 4 National Institute of Health, NHLBI, Bethesda, USA; 5 Baker IDI Heart and Diabetes Institute, Melbourne, Australia

## Abstract

Platelets can be found on the surface of inflamed and ruptured atherosclerotic plaques. Thus, targeting of activated platelets may allow for molecular imaging of vulnerable atherosclerotic lesions. We here investigated microbubbles (MB) functionalized with the selectin ligand sialyl Lewis^a^ individually (MB_sLea_) or dually with sLe^a^ and an antibody targeting ligand-induced binding sites of the activated GPIIb/IIIa receptor (MB_Dual_). Assessed by *in vitro* flow chamber, targeted MB exhibited increased adhesion to platelets as compared to MB_Control_. While MB_sLea_ rolled slowly on the platelets’ surface, MB_Dual_ enhanced the percentage of firm adhesion. *In vivo*, MB were investigated by ultrasound in a model of ferric chloride induced non-occlusive carotid artery thrombosis. MB_sLea_ and MB_Dual_ revealed a higher ultrasound mean acoustic intensity than MB_Control_ (p < 0.05), however MB_Dual_ demonstrated no additional increase in mean signal intensity as compared to MB_sLea_. The degree of carotid artery stenosis on histology correlated well with the ultrasound acoustic intensity of targeted MB (p < 0.05). While dual targeting of MB using fast binding carbohydrate polymers and specific antibodies is a promising strategy to support adhesion to activated platelets under arterial shear stress, these advantages seem not readily translatable to *in vivo* models.

## Introduction

While the degree of stenosis in coronary or cerebral vessels can be quantified by various established clinical imaging techniques, the vulnerability of atherosclerotic plaques and assessing their risk to rupture remain the holy grail in cardiovascular imaging^1,2^. Platelets play an important role in vascular inflammation and can promote plaque progression^3^. They can also be found on the surface of highly inflamed plaques and directly after plaque rupture, resulting in partial or even complete vascular occlusion and thereby stroke or myocardial infarction^4^.

Morphology of the vessel wall and atherosclerotic plaque formation of carotid arteries as imaged by regular ultrasound imaging can reflect both the risk for future cerebrovascular events and a given patient’s global vascular disease burden^5,6^. Since the degree of stenosis does not reflect vulnerability, it would be desirable to have an additional non-invasive modality for more detailed characterization. Molecular imaging offers the unique opportunity to produce contrast agents targeting specific cell types or cellular receptors^7^. For ultrasound imaging, a targeted imaging contrast agent usually consists of a micrometer-sized gas-filled microbubble conjugated to specific ligands such as antibodies, polymers or peptides^8^. We developed an antibody targeting the activated glycoprotein IIb/IIIa-receptor on platelets whereby ligand-induced binding sites (LIBS) become exposed after fibrinogen binds to the receptor^9^. Our previous molecular imaging studies demonstrated the successful use of this antibody for the targeted imaging of activated platelets in various disease^10–12^.

Since microbubbles measure up to 5 µm, targeted contrast agents are exposed to high shear stress both during initial capture and after adhesion to an endothelial molecular target. This applies in particular to imaging targets under high shear stress conditions, eg in large arterial vessels. In this respect, we demonstrated that the microbubble-targeting of P-selectin on platelets using the natural ligand sialyl Lewis^a^ leads to the capture and slow rolling of microbubbles on the endothelial surface, but no firm adhesion^13,14^. Thus, dual targeting using a first ligand with high on-rate such as sialyl Lewis and a second antibody-based ligand with a low off-rate could help improve the success of the target binding of microbubble contrast agents when imaging activated platelets.

For this purpose, we developed and validated a targeted microbubble (MB) with two ligands bound to the microbubble surface: an antibody against LIBS on activated platelets, and the selectin ligand sialyl Lewis^a^ polymer (sLe^a^). This construct was evaluated first *in vitro*, and thereafter applied to an *in vivo* ultrasound imaging application simulating a ruptured, non-occlusive plaque of the carotid artery in mice.

## Results

### Surface characterization of targeted microbubbles

**Figure 1:**
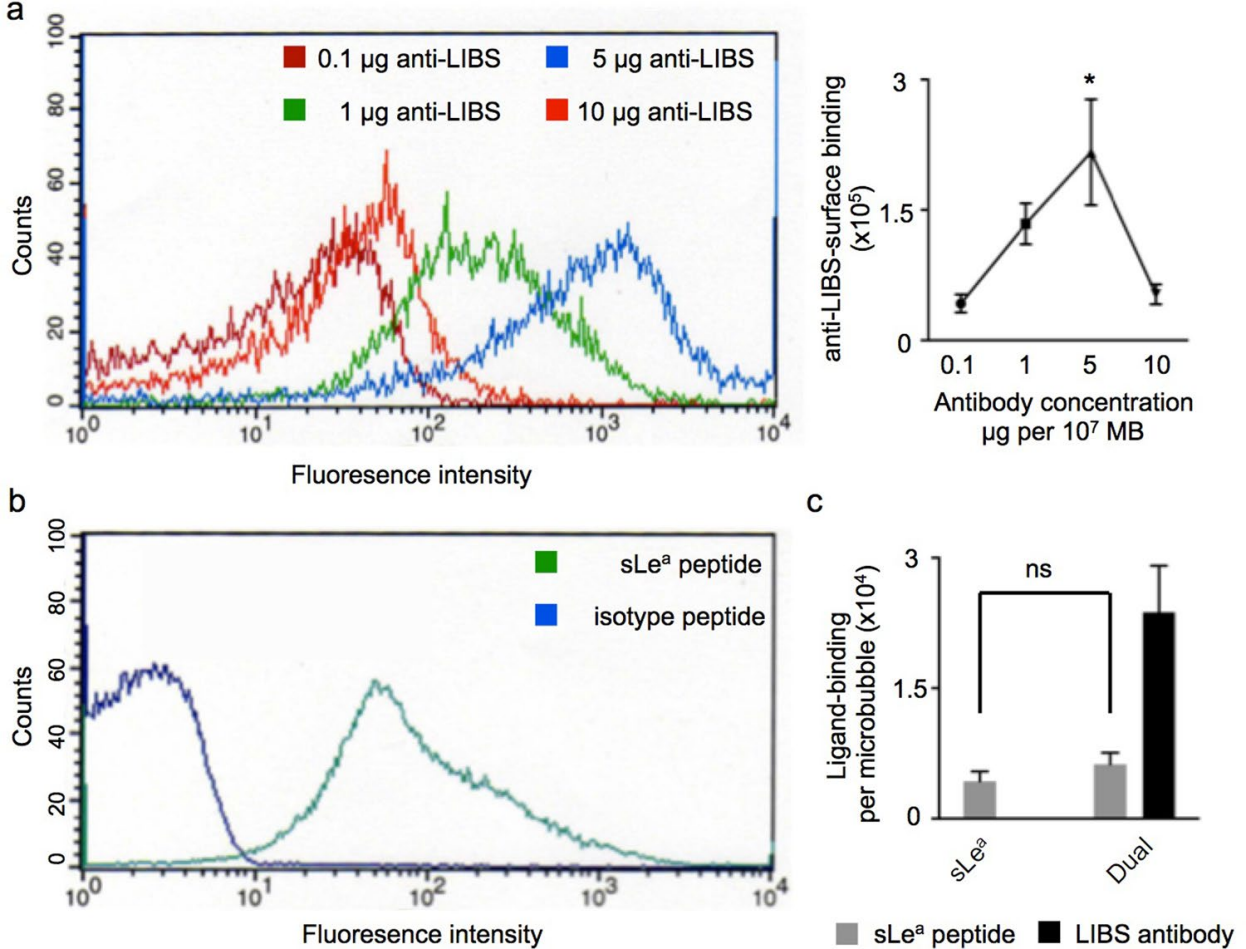
Functionalized targeted microbubbles (MB). (**a**) Surface-loading of targeted microbubbles (MB) with biotinylated anti-LIBS IgG-antibody at different concentrations 0.1, 1, 5 and 10 µg per 10^7^ MB. Flow**-**cytometric analysis of binding efficiency to MB and anti-IgG FITC antibody (left). Graphic illustration of anti-LIBS surface binding dependent on antibody concentration (right). *p < 0.05, n = 5 per group. (**b**) Flow**-**cytometric analysis of surface-integration of the sLe^a^-polymer using an FITC-labelled anti-sLe^a^ antibody compared to an isotype peptide. (**c**) Polymer-integration of sLe^a^ only (MB_sLea_) and dually labelled with sLe^a^ + anti-LIBS antibody (MB_Dual_) on the MB.

To optimize the surface-loading of targeted microbubbles with biotinylated anti-LIBS IgG-antibody, we analyzed their binding efficiency to MB using increasing concentrations of anti-LIBS antibody. Based on our results from fluorescence-based bead analysis, the greatest amount of surface loading of microbubbles with anti-LIBS antibody was achieved after incubating 5 µg anti-LIBS antibody per 10^7^ MB. Higher concentrations of anti-LIBS antibody (10 µg anti-LIBS per 10^7^ MB) reduced the surface loading (n = 9–10 per group, p < 0.05, One-way ANOVA Test; Fig. 1a).

Surface-integration of the sLe^a^-polymer was assessed via flow cytometry and a fluorescence-labelled anti-sLe^a^ antibody. Compared to control MB not bearing biotin-streptavidin-biotin bridges, incubating anti-sLe^a^ antibody with targeted MB resulted in a shift to the right of the fluorescence MB population indicating polymer-integration (Fig. 1b). Binding sites became saturated at sLe^a^ -polymer concentrations of 0.8 µg per 10^7^ MB. To investigate any interference between sLe^a^-polymer integration and anti-LIBS binding, we assessed MB_sLea_ before and after anti-LIBS conjugation. Anti-LIBS conjugation did not alter sLe^a^-binding (4209 sLe^a^/MB vs 4301 sLe^a^/MB, n = 10–14 per group, not significant, student-*t* test; Fig. 1c). However, the presence of sLe^a^ reduced anti-LIBS binding to the MB (2.1 × 10^5^ vs. 2.3 × 10^4^, n = 10–12 per group, p < 0.01, student-*t* test).

### Dual-targeted microbubbles improve firm binding in *in vitro* flow chamber

**Figure 2:**
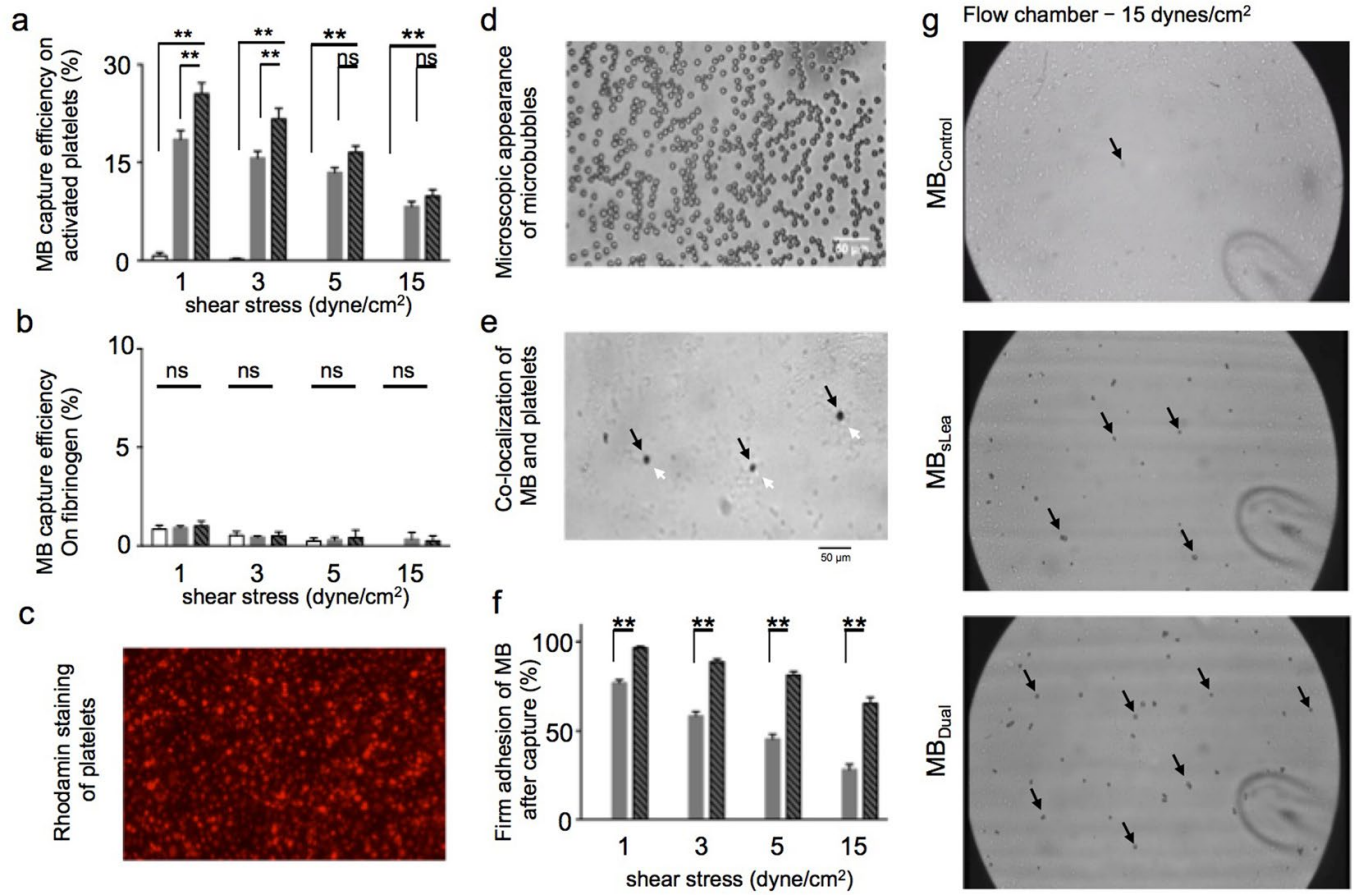
*In vitro* investigation of targeted MB. Assessment of capture efficiency of MB_Control_, MB_sLea_ and MB_Dual_ at increasing shear stress (1, 3, 5 and 15 dynes/cm^2^) on (**a**) activated platelets and (**b**) fibrinogen using a flow chamber set up. Capture efficiency was calculated as the ratio from MB-binding and MB-flux. (**c**) Rhodamin staining of platelets illustrates their distribution on a flow chamber plate after binding to fibrinogen coating (magnification 40x). (**d**) Macroscopic appearance of microbubbles. (**e**) Co-localization of MB (black arrows) and platelets (white arrows) on the flow chamber plate. (**f**) Assessment of firm adhesion of MB_sLea_ and MB_Dual_ at increasing shear stress (1, 3, 5 and 15 dyne/cm^2^) to activated platelets. Firm adhesion of MB was determined when an MB stopped rolling within the first 5 seconds after binding and remained stationary for at least 10 seconds. (**g**) Representative images from *in vitro* flow chamber. Equal concentrations of MB_Control_ (above), MB_sLea_ (middle) or MB_Dual_ (below) where flushed over a bed of activated platelets at a shear stress rate of 15 dynes/cm^2^. Increased firm binding of MB_dual_ leads to more MB bound to the surface covered with fibrinogen-activated platelets as compared to MB_sLea_ (arrows point to examples of MB on the surface). For motion pictures please see supplement movies. *p < 0.05, **p < 0.01, ns indicates “not significant”, n = 5 per group.

Using an *in vitro* flow chamber, we exposed MB to a layer of fibrinogen-activated platelets to investigate the binding properties of targeted MB under shear stress and to assess capture efficiency, defined as the binding of MB to platelets per flux. Compared to MB_Control_, sLe^a^-polymer increased MB capture efficiency significantly due to sLe^a^-mediated binding to platelets. Employing the dual targeting strategy with sLe^a^ and anti-LIBS (MB_Dual_) enabled us to further improve the capture efficiency of MB to activated platelets at low to intermediate shear stress rates. This effect became weaker at higher shear stress rates (5 and 15 dyne/cm^2^) (n = 9–13 per group, **p < 0.01, ns = not significant, one way ANOVA; Fig. 2a). To demonstrate the specificity of MB binding, we performed the same experiment without activated platelets. Running targeted MB over a fibrinogen layer at increasing degrees of shear stress resulted in unspecific capture efficiency of <1% for all microbubble species (n = 3–4 per group, not significant, one-way ANOVA; Fig. 2b).

We then assessed the quality of binding and the co-localization of platelets and MB in an *in vitro* flow chamber model (Fig. 2c–e). MB_Control_ did not relevantly bind to activated platelets and was not further quantified. MB_sLea_ resulted in binding, rolling, and firm adhesion to activated platelets. Firm adhesion was assumed if rolling platelets stopped rolling for at least 5 seconds. In comparison, firm MB arrest on the platelet layer was greater when using MB_Dual_. This effect became more pronounced with increasing shear stress (n = 24–28 per group, **p < 0.01, Mann Whitney-U test; Fig. 2f). Especially at shear rates of 5 and 15 dynes/cm^2^, use of MB_Dual_ led to a higher percentage of firm adhesion. Figure 2g illustrates representative images of the flow chamber experiment to visualize this effect.

### Targeted microbubbles enable the *in vivo* detection of vascular thrombosis

**Figure 3:**
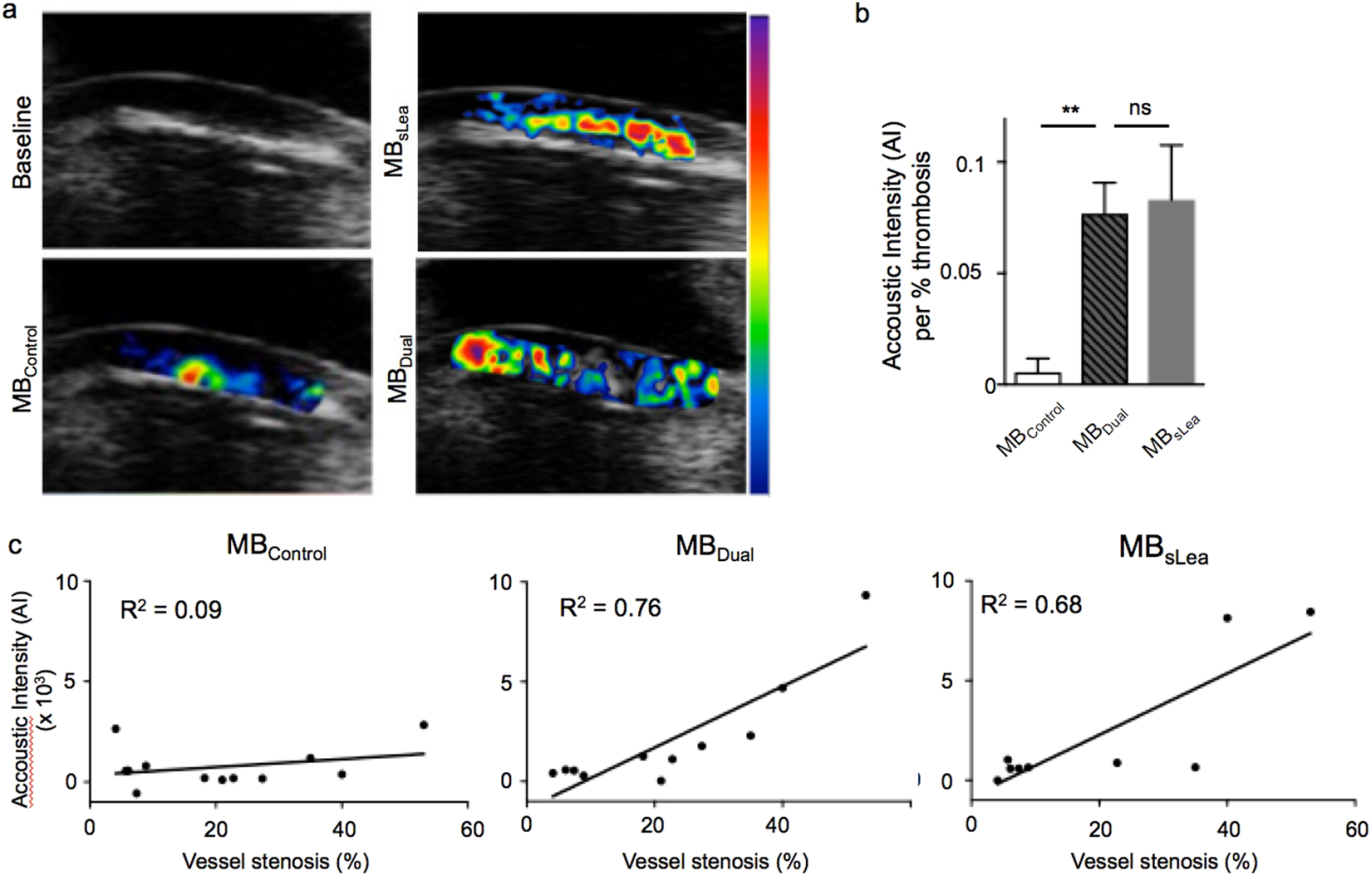
(**a**) *In vivo* ultrasound of the right common carotid artery after induction of a wall-adherent vessel thrombosis by ferric chloride. Baseline ultrasound image of the vessel before contrast injection (upper left), and color-coded images after injection of MB_Control_ (lower left), MB_sLea_ (upper right) or MB_Dual_ (lower right). Color-encoding reveals the area distribution of acoutic intensity (AI) of MB signal from low (blue) to high (purple) signal. (**b**) Comparison of mean AI per % thrombosis between MB_Control_, MB_Dual_ and MB_sLea_. (**c**) Correlation of AI with the degree of vessel thrombosis by histology after injection of MB_Control_, MB_Dual_ and MB_sLea_ **p < 0.01, ns indicates “not significant”.

We then took the dual-targeted MB approach to an *in vivo* model of ferric chloride-induced, non-occlusive vascular thrombosis. Using this method we evaluated specimens with 2–60% thrombotic vessel occlusion as confirmed by immunohistochemistry for platelet CD41 of excised vessels after ultrasound imaging. Figure 3a shows the right common carotid artery of a mouse after the induction of a non-occlusive thrombosis. The echo-rich signal from a paper mark helped us localize the area of interest. After injecting MB_Control_ (lower left), MB_sLea_ (upper right) or MB_Dual_ (lower right), the MB signal’s mean acoustic intensity was color-coded using a heat-map from blue (low mean acoustic intensity) to red/purple (high mean acoustic intensity). The coefficient of mean acoustic intensity per % thrombosis strongly increased in the area of vascular thrombosis after the injection of MB_Dual_ as compared to non-targeted MB_Control_ (n = 8–11 mice per group, MB_Control_ vs MB_Dual_: p < 0.01; MB_Dual_ vs MB_sLea_: not significant; one-way ANOVA; Fig. 3b). This increase in mean acoustic intensity in the vascular thrombotic region correlated well with the degree of vessel stenosis (MB_Control_: R^2^ = 0.09 vs. MB_sLea_: R^2^ = 0.68 vs. MB_Dual_: R^2^ = 0.76, n = 9–12 mice per group, Pearson correlation; Fig. 3c). However, the capture efficiency and increased firm adhesion of MB_Dual_ to activated platelets observed *in vitro* did not result in a significantly improved mean acoustic intensity of MB_Dual_ over MB_sLea,_ and only a minor change in the correlation between mean acoustic intensity and the degree of vessel stenosis as compared to *in vivo*.

## Discussion

Molecular imaging has evolved into an exciting tool that adds functional information to structural imaging, which may be especially useful in conjunction with cardiovascular diseases, whereby cellular or subcellular changes on the vessel surface may provide early evidence of vulnerability or thrombotic activity as the precursors of life-threatening cardiovascular events^15,16^. In recent years, molecular imaging has passed important hurdles to clinical translation but so far it is mainly restricted to distinct and expensive imaging techniques, eg magnetic resonance or nuclear imaging^17–19^. Targeted microbubbles (MB) have now set the stage for ultrasound molecular imaging as a widely available imaging technique, which could enable its broad application at less cost.

While ultrasound imaging is a standard procedure for assessing venous and arterial vessels, the high shear stress, especially in arterial vessels, remains a major challenge for molecular MB imaging. The relatively large size of MB as compared to their binding site exerts strong forces on the ligand-receptor interaction, thereby impairing target binding and thus sensitivity. Dual target strategies using complementary specific ligands have been successfully demonstrated to increase the capture of molecular imaging contrast agents in these settings^20,21^.

We therefore investigated a dually targeted ultrasound contrast agent towards activated platelets for detecting wall-adherent, non-occlusive carotid artery thrombosis.

We made our design to mimic the body’s physiological approach to capture circulating blood leukocytes under arterial shear stress conditions. Leukocytes first loosely bind to selectins to roll over the endothelial surface. After slowing down, adhesion molecules may bind firmly and arrest the leukocyte^22^. We hypothesized that, by combining the fast binding selectin ligand sialyl Lewis^a^ (sLe^a^) with the anti-LIBS antibody (which specifically binds to the activated glycoprotein IIb/IIIa-receptor), dual targeting would improve sensitivity and quality in the non-invasive detection of wall-adherent, non-occlusive carotid artery thrombosis via molecular ultrasound imaging.

To determine the optimal amount of anti-LIBS antibody and sLe^a^-polymer needed to ensure efficient saturation of MB binding sites, we first characterized the MB surface *in vitro*. As expected, increasing concentrations of anti-LIBS antibody correlated with anti-LIBS loading onto the MB surface. However, instead of surface saturation, high concentrations of anti-LIBS antibody (10 µg anti-LIBS per 10^8^ MB) led to a strong reduction in anti-LIBS surface loading on the MB. We concluded that this was most likely due to the antibody’s precipitation at high concentrations. sLe^a^ exhibited dose-independent surface loading at the concentrations we tested, but seemed to slightly reduce anti-LIBS antibody binding in the dual coupling approach. Steric interactions between the ligands cannot be excluded and must be acknowledged.

We then performed flow chamber experiments to prove this concept. Indeed, we noted that targeted MB (MB_sLea_ and MB_Dual_) increased capture and binding to activated platelets significantly more than unspecific MB_Control_ did at all shear stress rates. Capture efficiency was highest in MB_Dual_ at low to intermediate shear stress. As shown by Wang *et al*., the binding of anti-LIBS to activated platelets captures MB from the blood stream independently^23,24^. Therefore, sLe^a^ and anti-LIBS jointly improved capture efficiency.

After capture, dual targeting seemed to change the nature of MB binding. While captured MB_sLea_ slowly rolled over the surface of activated platelets, which is characteristic for selectin-mediated binding^22^, MB_dual_ adhered firmly more often and more persistently after rolling than did MB_sLea_ at all shear stress rates. The deceleration of MB via sLe^a^ on the flow chamber surface seems to help anti-LIBS to release more persistent target bonds to activated platelets, especially at high shear stress rates.

To investigate the translation of *in vitro* findings *in vivo*, we measured the mean acoustic intensity of MB_Control_, MB_sLea_ and MB_Dual_ in a murine model of non-occlusive carotid artery thrombosis via ultrasound imaging. As was the case *in vitro*, targeted MB exhibited a significantly stronger mean acoustic signal intensity than MB_Control_. We observed a good correlation between the degree of thrombosis and ultrasound mean acoustic intensity especially in conjunction with carotid stenoses of 2–60%. While dual targeted MB imaging provided a visually convincing working tool for detecting arterial thrombosis, we detected no incremental benefit from dual targeting with sLe^a^ and anti-LIBS over stand-alone sLe^a^ other than a subtle but not significantly improved correlation between mean acoustic intensity and the degree of vessel thrombosis. This finding gives us interesting insight into the binding dynamics of targeted MB under arterial flow. We noted that it was the MB capture from the blood pool rather than the quality of their binding that was decisive in determining the non-invasive imaging signal. It does not appear to be important whether the contrast agent is firmly bound onto the target structure or rolling on its surface. As the capture rate, especially at high shear stress rates (5–15 dyne/cm^2^), did not significantly differ between targeted MB groups, changes in overall binding of MB_Dual_ due to an increase in stationary MB at the vascular thrombus were not strong enough to affect the imaging signal’s mean acoustic intensity.

The unexpectedly strong (and in this setting specific) signal of sLea^a^ is a major limitation of our study. In more complex disease models like atherosclerotic plaque lesions, selectins may be expressed by both activated platelets and activated endothelium. This could dilute the apparent specificity of sLe^a^’s anti-platelet signal in our study^22^.

Furthermore, we did not investigate MB with anti-LIBS labelling only. Previous studies demonstrated that single anti-LIBS targeting is feasible for detecting vascular thrombosis via molecular imaging^10,12,24^, while the present study focused on synergistic effects of dual targeting.

## Conclusions

Non-invasive imaging of activated platelets on the surface of ruptured or inflamed atherosclerotic plaques is desirable to ensure the timely identification of a potential culprit lesion. Molecular ultrasound imaging constitutes a radiation-free and widely available technique for imaging cardiovascular pathologies from outside and inside the vessel.

In this study we demonstrated that the dual targeting of microbubbles with the selectin ligand siayl lewis^a^ and anti-LIBS led to greater capture efficiency, especially at low to intermediate shear stress and in conjunction with firmer adhesion of MB on activated platelets under all shear stress conditions *in vitro*. In contrast to our *in vitro* data, the advantages of MB_Dual_ were not readily transferred *in vivo*; they failed to induce significant changes in the signal as compared to more unspecific MB_sLea,_ while generally providing stronger contrast than MB_Control_. Further research will have to reevaluate dual target imaging and work towards the better detection of and prevention of cardiovascular events.

## Methods

### Animals

Female C57BL/6 N mice, age 10–12 weeks, were purchased from Charles River Laboratories (Sulzfeld, Germany). Mice were housed in cages containing 5 animals. Unlimited chow and water were provided. The numbers of animals used for each study group are indicated in the figure legends. All experimental protocols were approved by the Ethics Committee of Freiburg University and the regional council of Freiburg, Baden-Wuerttemberg, Germany: license number 35-9185.81/G-09/47). Experiments were conducted in accordance with FELASA, GV-SOLAS standards for animal welfare.

### Ligands

Two different ligands were linked to microbubbles (MB), either to prepare single targeted MB (with only one of the ligands on the MB surface; MB_sLea_) or to dually target MB (with both ligands on the MB surface; MB_dual_).

The first ligand was the fast binding ligand sialyl Lewis^a^ polymer (sLe^a^) in a biotinylated formulation (Glychotech, USA). sLe^a^ polymer has a molecular weight of approximately 30 kDa, carrying a functional tetrasaccharide group attaching to E- and P-selectin.

The second ligand was an IgG-antibody selectively binding to ligand-induced binding sites (LIBS) of the activated GPIIb/IIIa receptor on platelets^9^. This antibody is generated from hybridoma cells and has been used in molecular imaging studies to detect activated platelets^25^. After purification, the antibody was biotinylated, and successful biotinylation was demonstrated by performing a commercially available HABA assay (2-(4′-hydroxyazobenzene) benzoic acid, Thermo Scientific, Rockford, USA). Protein concentration was assessed by a bicinchoninic acid assay (BCA assay, Thermo Scientific).

### Microbubble preparation

Lipid-shelled biotinylated decaflourobutane MB were prepared as described elsewhere^26^. Briefly, MB were prepared by sonifying a solution containing distearoylphosphatidylcholine (2 mg/ml), polyethylenglycolstearate (1 mg/ml) and biotinylated polyethylenglycol-distearoylphosphatidylethanolamine (PEG3400-DSPE). After centrifugal washing, the MBs were characterized for mean diameter and size distribution using a Coulter Multisizer (Beckman Coulter). Biotin-streptavidin-biotin bridging was then done as previously described^13^ to attach the ligands to the microbubble surface. To prepare MB carrying the sialyl Lewis^a^ polymer (MB_sLea_), 10^7^ MB carrying streptavidin were incubated in 0.8 µg sLe^a^ as described previously^13^. To prepare MB carrying an antibody to ligand-induced binding sites (LIBS) of the activated GPIIb/IIIa receptor (MB_LIBS_), 10^7^ streptavidin-labeled MB were incubated in 0.1 µg, 1 µg, 5 µg, or 10 µg of the LIBS-Ab. We incubated with different amounts of LIBS-Ab to determine the amount of antibody needed for optimal antibody-binding to the MB surface^20^.

Dual targeted MBs were first incubated in the sLe^a^-polymer and, after 10 minutes of incubation time, the LIBS-antibody (5 µg/10^7^MB) was added according to our flow cytometry results. After incubation in both ligands, MBs were washed several times to remove unbound ligands. Structural integrity and aggregation of MB were assessed microscopically or using a Coulter Multisizer. Control MB (MB_Ctr_) consisted of streptavidinized MB covered with similar concentrations of unspecific IgG and a polymer of equal size to the used sLe^a^-polymer, but without active tetrasaccharide groups.

### Platelet preparation

After approval by the University of Freiburg’s Ethics Committee, citrated blood was taken from healthy volunteers not taking any medications (especially platelet inhibitors), and centrifuged with 150 g for 10 minutes (Multifuge 3 s, Heraeus Instruments, Osterode, Germany). Platelet-rich plasma (PRP) was obtained by centrifuging heparinized blood for 10 minutes at 100 g. Cells were diluted 1:20 with PBS without calcium/magnesium to a final concentration of 250.000 per µl. In the following sections‚ ‘PBS’ stands for phosphate buffered saline without calcium and magnesium, otherwise it is mentioned if the PBS used contained calcium and magnesium.

### Quantification of ligands on the microbubble surface

Surface binding of sLe^a^ polymer or LIBS-Ab was assessed by flow cytometry using beads with specific antibody binding capacity for calibration (Kit Quantum™ Simply Cellular® anti-Mouse IgG (Bangs Laboratories, Fishers, USA). To establish a calibration curve for LIBS-antibody, beads were incubated in 30 µg LIBS antibody/10^7^ beads and 2 µg10^7^ beads FITC anti-IgG secondary. For sLe^a^-polymer, beads were incubated with 0.8 µg/10^7^ beads sLea polymer followed by 1 µg/10^7^ beads PE-conjugated anti-sLe^a^ (anti-CA19-9) antibody. Targeted MBs were counted by fluorescence (10^7^ events). Baseline fluorescence was acquired using MB stained with FITC- or PE- labeled IgG isotype-control antibody. Each flow cytometry preparation contained 10^7^ MB targeted with LIBS-antibody, sLe^a^-polymer or both, as described above. Targeted MB were diluted with 500 µl PBS to obtain a sufficient sample volume. All the following incubation steps were taken using a perflourobutane-gas headspace to prevent microbubble gas loss. Calibration curves were run immediately prior to labelled MB. Antibody surface concentration was assessed using an excel-based program provided by the manufacturer.

### Flow Chamber

The flow chamber setting was created as previously described with an inverted flow deck and specific rectangular flow path^13,20^. 35 mm polystyrene cell culture wells (Fa. Corning Incorporated, Corning, USA) were covered with fibrinogen and incubated over night at +4 °C. After washing wells with PBS, blocking with casein (Blocker^TM^ Casein in TBS, Fa. Thermo Scientific, Rockford, USA) removed unspecific surface epitopes. Thereafter, activated platelets from platelet rich plasma were incubated in these wells for 30 min at +37 °C. Platelets were activated using 20 µM ADP (Fa. MöLab, Langenfeld, Germany). Presence of platelet coverage was confirmed via light microscopy.

Wall shear stress was indicated in dynes/cm^2^ based on the equation τ = 6µQ/bh_2_, where τ is wall stress (N/m^2^), µ is fluid viscosity (0.001 N/s^2^), Q is flow rate (m^3^/s) and b;h are measurements of the chamber width and height (m). Flow chamber experiments were conducted in an inverse position to account for MB buoyancy. MB formulations were dispersed in 6 ml of Dulbecco’s PBS using a continuous magnetic stirrer. Using a syringe pump (Harvard Apparatus, Holliston, Massachusetts, USA) MB formulations were passed through the flow chamber at decreasing shear stress rates: 15, 10, 5, 3 and 1 dynes/cm^2^. Higher shear stress rates were not investigated due to insufficient overall adhesion of microbubbles. A microscope with CCD-camera (Horn, Aalen, Germany) mounted onto the center of the flow chamber transferred real time images with a field of view of 420 × 350 µm, enabling the quantification of capture efficiency, which was then calculated as the ratio of MB binding and flux. Furthermore, MBs rolling on the surface were distinguished from firmly binding MBs. Firm adhesion was determined when an MB stopped rolling within the first 5 seconds after binding and remained stationary for at least 10 seconds.

### Induction of carotid artery thrombosis and animal instrumentation

To create a wall-adherent platelet-derived thrombosis in the carotid artery simulating a ruptured plaque, we applied a modification of the well-established model of ferric chloride-induced carotid artery thrombosis^27^. Reproducible creation of a non-occlusive, wall-adherent thrombosis with residual blood circulation and therefore contrast agent flow over the surface has been also described by our group^28^. In brief, 10–11-week-old male C57BL/6 mice weighting 24+/− 2 g (Charles River, Germany) were used. Mice were anesthetized via injection with ketamine i.p. (100 mg/kg BW) and xylazine (5 mg/kg BW) and were then placed under a dissecting microscope. An incision in the skin was made superficial to the right common carotid artery. The fascia was dissected and a segment of the right common carotid artery exposed. Thrombosis was induced by applying a piece of filter paper (1 × 2 mm, GB003, Schleicher & Schuell) saturated with ferric chloride (6,5% solution, Sigma) under the right carotid artery, which was removed after 3 min. Furthermore, a jugular vein catheter was placed on the contralateral side for injecting the contrast agents. The animals were transferred to a temperature-controlled imaging platform (Vevo Imaging Station) and the core temperature was maintained at 37 °C.

### *In vivo* ultrasound imaging

Contrast enhanced ultrasound molecular imaging was performed with a Sequoia Acuson C512 (Siemens Medical Systems) equipped with a high-frequency linear-array probe (15L8). The probe was placed over the exposed carotid artery and held in place with a railed gantry system. Color Doppler imaging was applied to align the carotid artery parallel to the image plane. To detect the microbubble contrast agents, we carried out power modulation and pulse inversion imaging (Cadence^TM^ contrast pulse sequencing, CPS) at 7 MHz and a dynamic range of 50 dB. The gain settings were adjusted to avoid visible speckle and held constant throughout the experiment. MB_Control_, MB_sLea_ and MB_Dual_ (3 × 10^6^ microbubbles per injection in 150 µl PBS each) were injected into each mouse in random order. Ultrasound imaging was halted from the injection until 8 minutes later. Imaging was then resumed at a mechanical index of 0.87 with a time trigger set to acquire an image frame every 500ms. The first image frame acquired was used to derive the signal from the total amount of microbubbles present in the carotid artery. The microbubbles in the ultrasound beam were then destroyed with several (>20) imaging frames at a decreased (40 ms) trigger interval. After their destruction, another set of image frames at a trigger interval of 500ms was acquired to derive the signal from freely circulating microbubbles. We then digitally subtracted the post-destruction images from the first image frame to derive the signal from attached microbubbles only.

### Histology and quantification of stenosis

After ultrasound imaging, animals were terminally anesthetized using ketamine and xyalazine. The vascular system was flushed with saline via the left ventricle, then the carotid artery containing the thrombus was removed. Samples were embedded in OCT TissueTec (Sakura Finetec, Netherlands) and snap frozen for histology.

To detect and quantify wall-adherent thromboses histologically, mouse platelets were stained using a rat anti-mouse glycoprotein IIb (CD41) polyclonal antibody (Clone MWReg30, GeneTex, USA), as described previously^12^. Using avidin-peroxidase staining after incubation with a biotinylated secondary antibody (biotinylated anti-rat IgG, Vector Laboratories, Burlingame, USA) and Vector Red (Vector Laboratories, Burlingame, USA) enabled sensitive visualization of the primary antibody binding sites. For each animal, 12 representative sections were chosen and the degree of thrombosis quantified in percent of the total vessel lumen.

### Statistical analysis

Statistical analysis was performed using GraphPad Prism software (GraphPad Software, Inc. USA). Data were expressed as mean ± SEM and statistical analysis was performed by using a two-sided student’s *t*-test if the pre-test for normality (D’Agostino-Pearson normality test) was not rejected at 0.05 level, otherwise a Mann-Whitney U test for nonparametric data was applied. One-way ANOVA-analysis followed by a Bonferroni post-test was used for the comparison of more than two groups where applicable. Values p < 0.05 were reported as statistically significant.

## Electronic supplementary material

Supplementary movie 1

Supplementary movie 2

Supplementary movie 3
